# Parents’ experiences of being involved in medical decision-making for their child with a life-limiting condition: A systematic review with narrative synthesis

**DOI:** 10.1177/02692163231214414

**Published:** 2023-12-06

**Authors:** Kristyna Polakova, Faraz Ahmed, Karolina Vlckova, Sarah G Brearley

**Affiliations:** 1 Division of Health Research, Faculty of Health and Medicine, Lancaster University, Lancaster, UK; 2 Center for Palliative Care, Prague, Czech Republic

**Keywords:** Parents, life experience, decision making, child, palliative care, life-limiting

## Abstract

**Background:**

Parental involvement in the decision-making processes about medical treatment for children with life-limiting conditions is recognised as good practice. Previous research highlighted factors affecting the decision-making process, but little is known about how parents experience their participation.

**Aim:**

To explore how parents experience their participation in the process of decision-making about treatment and future care for their children with life-limiting conditions.

**Design:**

A systematically constructed review using narrative synthesis. The PRISMA guidelines were followed to report the findings. Databases Medline, EMBASE, SCOPUS, CINAHL and PsycINFO were searched up to December 2023. The study protocol was registered at PROSPERO (RN CRD42021215863).

**Results:**

From the initial 2512 citations identified, 28 papers met the inclusion criteria and were included in the review. A wide range of medical decisions was identified; stopping general or life-sustaining treatment was most frequent. Narrative synthesis revealed six themes: (1) Temporal aspects affecting the experience with decision-making; (2) Losing control of the situation; (3) Transferring the power to decide to doctors; (4) To be a ‘good’ parent and protect the child; (5) The emotional state of parents and (6) Sources of support to alleviate the parental experience.

**Conclusions:**

Parental experiences with decision-making are complex and multifactorial. Parents’ ability to effectively participate in the process is limited, as they are not empowered to do so and the circumstances in which the decisions are taking place are challenging. Healthcare professionals need to support parental involvement in an effective way instead of just formally asking them to participate.

**What is already known about the topic?**

- Parents of children with life-limiting conditions are required to make complex and challenging medical decisions about medical care for their child.
- The ability of parents to engage in decision-making is affected by several factors.
- The knowledge of how parents experience their participation is limited.

**What this paper adds?**

- Participation in the decision-making process is an emotionally challenging situation and parents experience a wide range of negative emotions.
- Parents’ ability to make decisions for their child is affected by their emotional state and their perceived lack of confidence to act on behalf of their child caused by limited medical knowledge, emotional exhaustion and insecurities.
- Making difficult decisions in challenging circumstances can result in difficulties in maintaining the parental role and in losing the ability to make decisions for their child.

**Implications for practice, theory or policy**

- Parental negative experience can be mitigated by a sensitive attitude of the clinicians, providing parents with adequate support and preparing them for decision-making.
- Parents should be actively invited and encouraged to participate in the decision-making by clinicians, but it is necessary to tailor the level of participation individually for each parent and enable them to engage at their preferred level.
- Further research should focus on the experience of fathers and single parents, as this population is understudied.

## Background

The involvement of parents in the medical decision-making process is seen as a standard practice in modern paediatric medicine.^1,2^ Individual needs and preferences of each parent should be acknowledged as the level at which parents want to be included may differ.^3,4^ This also applies to parents of children with life-limiting or life-threatening conditions.

Conditions which can be classified as life-limiting or life-threatening represent a diverse group of often rare diagnoses, but together they affect a large population of children, with a worldwide estimation of around 21 million children.^5^ Life-limiting and life-threatening conditions can be divided into four categories, based on the course of the illness and the expected outcome: (I) life-threatening conditions with possible cure which can fail, such as cancer; (II) conditions with inevitable premature death where intensive treatment prolonging life is available, such as cystic fibrosis; (III) progressive conditions without curative treatment options, where treatment is exclusively palliative, for example Batten disease and (IV) irreversible but non-progressive conditions causing severe disability and likelihood of premature death like cerebral palsy.^2^ Although conditions which fall within the life-threatening category can be possibly curable, they can also be fatal and result in premature death of the ill child^6^; therefore, for this review, the term life-limiting conditions will be used for all four categories. All conditions which fall within the categories presented above are characterised by uncertain prognoses and unpredictable changes in a child’s health.^7,8^ Therefore parents have to make complex and often challenging decisions about medical care during the child’s life.^9,10^

Within the population of parents of children with life-limiting conditions, the available evidence suggests a strong preference for active parental participation in decision-making.^8,10^ The ability of parents to engage in decision-making for their child is affected by several aspects. The most highlighted aspect is concerning the child’s quality of life,^7,11,12^ followed by having a sufficient amount of information and sensitive communication with healthcare professionals.^10,12–14^ Additionally, parents need adequate support from clinicians, who act as gatekeepers in the decision-making process^15,16^ to be able to actively participate.^7,10,12^ During the decision-making process, healthcare professionals and parents should work together as partners and reach the decision through discussion.^17,18^ To give parents adequate support during this process, it is important to understand how parents experience their participation, but this knowledge is limited. Available systematic reviews in this area have focussed on exploring factors affecting the decision-making process, parents’ perception of their role or the level of their involvement,^7,8,10,12,19–21^ or their experience with end-of-life care.^22–25^ Furthermore, the available studies tend to distinguish between the four categories of life-limiting conditions, either focussing on parents of children with cancer^12,23,25^ or children with complex healthcare needs and disabilities,^7,8^ despite evidence that parental experiences of caring for of their child are similar irrespective of the child’s condition.^26^ Distinguishing among the four categories of life-limiting conditions can hinder the identification of possible similarities in the experience of making decisions about medical care and thus limit our understanding of this phenomenon.^7,10^ By bringing together studies exploring parental experience with decision-making for children regardless of their condition, it is possible to fill the gap in the available literature and to gain a better understanding of the decision-making process. The need for research focussed on communication between healthcare professionals and parents, including care-related decision-making, was identified among research priorities within the population of children with life-limiting conditions.^27^

Therefore, the purpose of this systematic review was to identify and synthesise available literature exploring how parents experience their participation in the process of decision-making about treatment and future care for their children with life-limiting conditions.

## Aim

A systematic review of the literature to explore how parents experience their participation in the process of decision-making about treatment and future care for their children with life-limiting conditions. The review question is: what are the parental experiences of the decision-making process for children with life-limiting conditions?

## Methods

The presented systematic review utilised the guidance for narrative synthesis by Popay et al.^28^ Narrative synthesis enables the integration of different types of evidence, including qualitative and quantitative data,^29^ permitting data from different types of studies to be collated into a homogenous group, while also identifying any differences in the studies and gaps in the literature.^30^

The review was reported by using the Preferred Items for Systematic Reviews and Meta-Analysis (PRISMA) guidelines^31^ (Supplemental Appendix 1) and registered at PROSPERO on 12 February 2021 (registration number: CRD42021215863).

### Inclusion criteria

Following inclusion and exclusion criteria were applied to each study (see Table 1).

**Table 1.** Inclusion and exclusion criteria.

Inclusion criteria	Exclusion criteria
Parents/legal guardians, including bereaved parents of children 0–19 years old diagnosed with a life-limiting condition	Studies including parents/legal guardians of children with life-limiting conditions older than 19 years at the time of the study
	Studies focussed on parental decisions made before the birth of a child diagnosed with a life-limiting condition before birth
	Studies focussed on the experience of parents of prematurely born babies and parents with newborn babies <28 days old
Reports on primary experience of parents/legal guardians involved in the decision-making process about the care of their child	Studies that do not report on the parental experience from the parents’ perspective and accounts of parental experience obtained from other participants involved in the decision-making process (such as doctors and nurses)
Studies reporting on parental experience with decision-making about healthcare for their child	Studies reporting on experience with phenomena other than decision-making in healthcare, including care experience, the experience of siblings, experience with providing care at home, care transition, decisions regarding fertility options for cancer patients and organ donation
English or Czech language	Other languages
Reports on primary findings of qualitative, quantitative or mixed methods research. Published in peer-reviewed journal	Commentaries, editorials, opinion papers, secondary data analysis, review articles, conference abstracts and case studies including just/only one case. Any study published in non-peer-reviewed journals.
Published between 2000 and 2023	Studies published before 2000

### Information sources and search strategy

The literature search was conducted in Medline, EMBASE, SCOPUS, CINAHL and PsycINFO in December 2020. The search terms were developed together with a subject librarian, and MeSH terms were used to enhance the search strategy. Details of the search strategy used in Medline database are presented in Table 2. Hand searching of the key journals was used in The Journal of Pediatrics, Journal of Pediatric Nursing, Journal of Hospice and Palliative Nursing, Palliative Medicine and MDPI Children. To identify any potentially relevant studies, included papers were checked for citation tracking. The searching process was documented by using the PRISMA 2020 statement: an updated guideline for reporting systematic reviews.^31^

**Table 2.** Search concepts for MEDLINE database.

Concept number	SPIDER	Pearl growing	MeSH	Search query
Concept #1	Parent Guardian	Caregiver	Parents Mothers Fathers	Parent* OR mother* OR father* OR guardian OR caregiver
Concept #2	Decision	Decision support	Decision making	decision OR decision making OR decision support
Concept #3	Experience	Perception View Feeling Attitude Belief	Life experience	experience OR view OR feeling OR perception OR attitude OR belief*
Concept #4	Child	Infant	Children Paediatric	child* OR infant OR paediatric
Concept #5	Life-limiting Life-threatening	Medically complex	Disabled Severely disabled Cancer Oncology Neoplasm Tumour Intensive care Long term care Terminal care	‘life-limiting’ OR ‘medically complex’ OR disabled OR ‘severely disabled’ OR ‘terminal care’ OR ‘long term care’ OR ‘intensive care’ OR cancer* OR oncolog* OR tumour* OR tumour* neoplasm OR malignan*

### Study selection

All identified papers were processed by the management tool EndNoteX9. Duplicates were removed electronically and manually. Titles and abstracts were screened independently against the inclusion criteria, and studies which met the inclusion criteria were read in full text by KP and KV. Any disagreement was resolved with SB and FA.

### Data collection and synthesis

Data from the included studies were extracted using NVivo software. Additional data were extracted in Excel and Word. The narrative synthesis was conducted by KP and subsequently reviewed by SB and FA. During the first stage of the narrative synthesis,^28^ each included study was analysed separately, and a textual description of the parental experience was developed. From each study, the direct citations from parents describing their experience with decision-making were extracted using NVivo. The description of parental experience presented by the study authors was also included in the synthesis. The data synthesis process included categorising the studies based on their setting (oncology and life-limiting) and participants (mothers and fathers) to allow comparison of the experiences with decision-making. This process was followed by data analysis using an open coding approach. Inductive codes identifying parental experience with the studied phenomenon were developed and subsequently collated together based on their similarities, thus developing preliminary themes used as a matrix during the analysis. The coding process included merging codes together, re-coding and developing new themes and subthemes. The data extraction and analysis were done by KP, identified themes were developed in consultation with SB and FA. Six themes were developed and are presented in the Results section.

### Data evaluation

With the aim to include only studies of a sufficient methodological rigour all of the included studies were evaluated using a quality assessment tool developed for critical appraisal of studies with different phenomenological backgrounds.^32^ This tool was previously used to assess the quality of systematic reviews in palliative care settings.^33,34^ The Hawker et at. tool^32^ evaluates nine components the score for each component ranges between 1 and 4; the overall minimum score is 9, the maximum is 36, which denotes high quality of the study. To assess the overall quality of the included studies the following grades definitions were used: high quality, 30–36 points; medium quality, 24–29 points and low quality, 9–24 points. In previous systematic review which used the Hawker et al. tool the minimum score for including studies was set at score of 20.^33^

Quality assessment was completed independently (by KP and KV), final scores were appointed after comparing individual scores and through discussion of possible differences. The assessed studies had scores between 26 and 36, with a median score of 32, which was considered as medium or high quality. Therefore, all eligible studies were included in the final synthesis.

## Results

**Figure 1.**
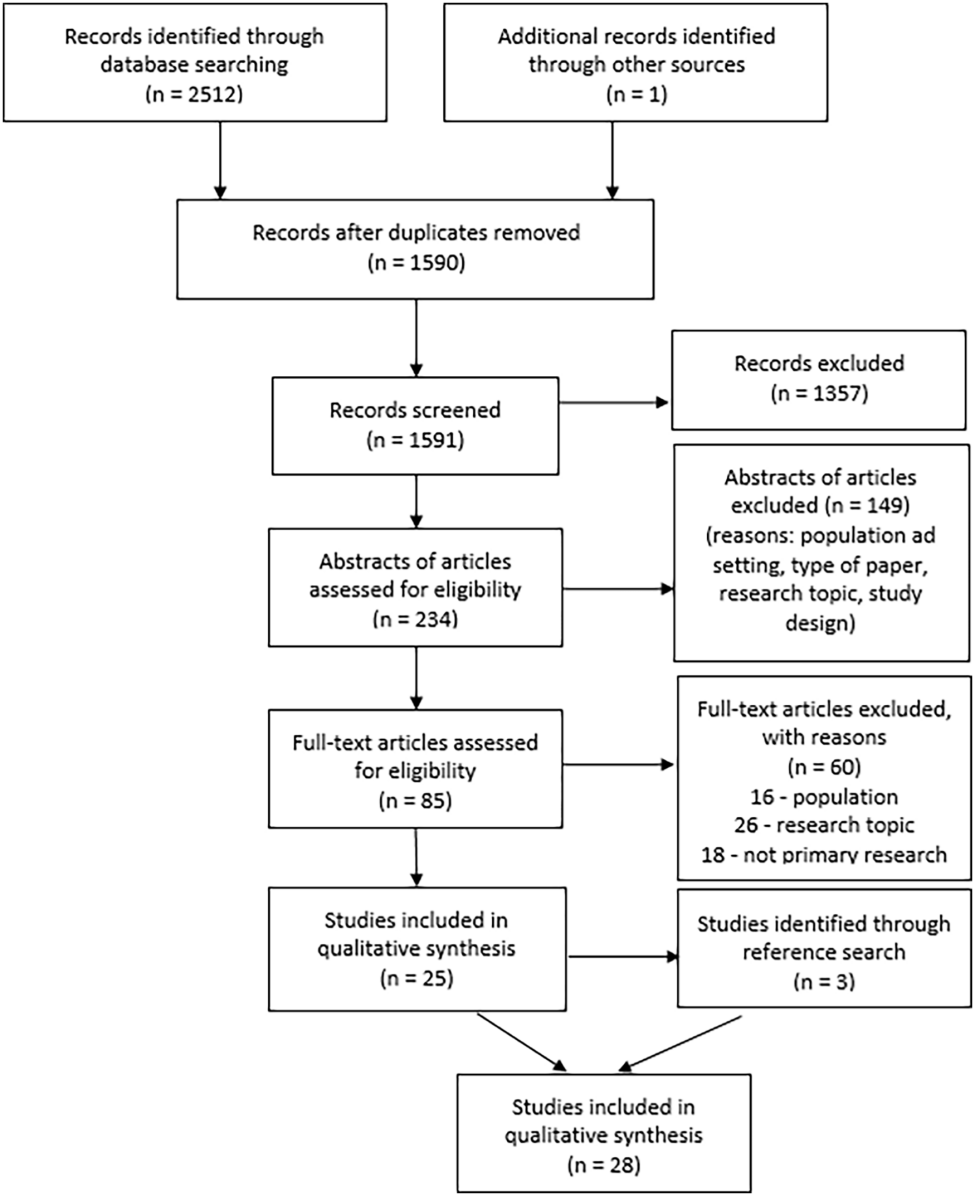
PRISMA flow diagram.^22^

After deduplication, 1591 studies were screened for eligibility using titles and abstracts. A total of 85 papers were read in full, with 25 meeting the inclusion criteria. Three additional studies were identified through citation tracking, resulting in 28 papers being included in this systematic review (see details in Figure 1).

## Overview of the studies

The methodological design of the included studies was mainly qualitative (*n* = 25), with three mixed methods studies.^35–37^ The included papers originated from 15 countries (see Table 3) and were mostly published between 2010 and 2023 (*n* = 26); two studies were published in 2005 and 2007.^38,39^ A total of 13 studies were set exclusively at oncology setting,^35,37,40–50^ 12 studies explored the decision-making of parents with children with life-limiting conditions^36,38,51–60^ and 3 had a population with mixed diagnoses.^39,61,62^ Six studies were set in intensive care units.^38,39,53,55,57,61^

**Table 3.** Overview of the included studies.

Author, year, country	Method/data collection	Objective/aim of the study	Setting	Quality score	Participants	Type of decision	Key findings
Atout et al. (2017), Jordan	Qualitative study Participant observation and semi-structured interviews	To understand the experiences of mothers of children with palliative care needs about their involvement in decision-making.	Life-limiting conditions	32	15 mothers 12 physicians 20 nurses	Treatment and care (not closely specified)	When making decisions, mothers relied on doctor’s expertise. They were experiencing a lack of confidence to make their decisions. Mothers were worried they would feel guilty in the future if making the decision for their children.
Badarau et al. (2017), Switzerland Romania	Qualitative study Interviews	To examine the perspectives of parents of children with cancer and their physicians on the experiences with participation in decision-making.	Oncology	32	37 parents (29 mothers, 5 fathers and 3 grandmothers) 26 physicians	Starting treatment Trial treatment Fertility treatment Treatment and care (not closely specified)	Participants in both countries described decision-making processes in similar ways. Parents could only participate in less important decisions.
Bandinelli, Goldim (2016), Brazil	Mixed-method study Semi-structured interviews and questionnaires	To get an understanding of the decision-making phenomenon from the perspectives of parents.	Oncology	32	10 parents (9 mothers and 1 father)	Starting treatment Catheter insertion	Parents felt like they did not have a real choice. They experienced a lack of time to make the decision and felt anxiety and fear. Parents found it difficult to grasp the reality of the diagnosis and act on it.
Beecham et al. (2016), UK	Qualitative study In-depth interviews	To increase the understanding of how parents approach and experience advanced care planning for their children.	Life-limiting conditions	32	18 parents (including 9 bereaved parents, 16 mothers and 2 fathers)	Place of care Place of death Limitation of treatment	Parents wanted to keep options open and to be able to change their minds. It was difficult for parents to foresee the possible consequences of treatment limitation. Making decisions about future treatment options was difficult as parents perceived the options as hypothetical.
Bergviken, Nilsson (2019), Sweden	Qualitative study Interviews	To explore how parents of children with cancer choose which type of central access device to use.	Oncology	32	17 parents (11 mothers and 6 fathers)	Central access device	Parents were making difficult decisions in a limited amount of time and in stressful situation. They were not sure which type of central access device was the right one and had difficulty to foresee the future.
Bogetz et al. (2022), USA	Qualitative study Semi-structured interviews	To explore the parental experience with decision-making for children with severe neurological impairment.	Life-limiting conditions Intensive care unit	26	25 parents (19 mothers and 6 fathers)	Treatment and care (not closely specified)	Parents acted as advocates for their children to ensure they would get the treatment they needed; parents felt they were not listened to by the medical team. When making decisions, parents felt responsible for the outcome while uncertain if the treatment would benefit the child.
Carlisle et al. (2022), USA	Qualitative study Semi-structured interviews	To get an understanding of parental preferences for surgical counselling when deciding on surgery for solid tumours.	Oncology	27	10 parents (gender not specified)	Aggressive treatment Tumour resection	Parents preferred to be involved in the decision-making process, but in some cases, parents did not have enough information and were not adequately engaged. Parents acted as advocates for their children. Participation in decision-making was experienced as overwhelming and frustrating if they felt not included. Parents found it difficult to ask the surgeons questions as they did not know what to ask.
Carnevale et al. (2007), Canada, France	Qualitative study Semi-structured interviews	To examine whether physicians or parents assumed responsibility for treatment decisions and how this related to the parental experience.	Life-limiting conditions Oncology Intensive care unit	32	31 parents (19 mothers and 12 fathers) 9 physicians 13 nurses	Life support Surgical interventions	Parents described their experience as very hard. They felt like they were abandoning their child if they agreed to stop treatment. Parents found it difficult to concentrate as they were in a state of shock.
Carnevale et al. (2011), Italy	Qualitative study Focus groups and interviews	To explore how life-sustaining treatment decisions were made for critically ill children in Italy and how were these decisional processes experienced by physicians, nurses and parents.	Life-limiting conditions Intensive care unit	26	9 parents (including 6 bereaved parents, 7 mothers and 2 fathers) 16 physicians 26 nurses	Life-sustaining treatment (not closely specified)	Parents found it difficult to make decisions, and they relied on the physician’s advice. They had difficulty processing all the information due to their emotional state.
De Clerq et al. (2022), Switzerland	Qualitative study Semi-structured interviews	To get an understanding of how parents experienced decision-making about initiating treatment for their deceased children diagnosed with diffuse intrinsic pontine glioma.	Oncology	30	25 bereaved parents (14 mothers and 11 fathers)	Starting cancer treatment, including radiotherapy and chemotherapy	Parents felt there were no viable options due to the lack of treatment protocols. Parents knew the condition was terminal, but they hoped for a miracle. At the same time, they focussed on the child's quality of life. Some parents decided to pursue the treatment to get additional time with the child, while others tried alternative therapies. Parents felt at peace with their decisions. Some found support in faith.
Edwards et al. (2020), USA	Qualitative study Semi-structured interviews	To explore the parental experience of decision-making to initiate long-term ventilation.	Life-limiting conditions	31	44 parents (34 mothers and 10 fathers)	Initiation of long-term ventilation	Parental experience with making decisions for the initiation of long-term ventilation was described as extremely difficult. They felt like they did not have a real choice and questioned the quality of the child’s life. They could not comprehend what long-term ventilation meant for everyday life.
Graetz et. al. (2022), Guatemala	Mixed-methods study Semi-structured interviews and cross-sectional survey	To explore the decision-making preferences and experiences of parents of children with cancer.	Oncology	29	118 parents (89 mothers and 29 fathers) 1 grandparent 1 sibling	Treatment and care (not closely specified)	Parents preferred the healthcare providers would make decisions about treatment as they trusted their medical opinion. Most parents (64%) did not regret their decisions.About 24% of parents asked for advice from religious or spiritual leaders.
Gurková et al. (2015), Slovakia	Qualitative study Semi-structured in-depth interview	To illuminate and analyse the experience of parents of children with failed cancer treatment and the death of their child.	Oncology	29	5 bereaved parents (4 mothers and 1 father)	Starting treatment Limitation of treatment Trial treatment Aggressive treatment Bone-marrow transplant	Parents felt like being forced into a decision or being in conflict with the doctors. They lacked support and understanding from healthcare professionals and acted as advocates for their children.
Huang et. al. (2022), Taiwan	Qualitative phenomenological study In-depth interviews	To explore the lived experience of parents of children with brain tumour during decision-making.	Oncology	33	10 parents (7 mothers and 3 fathers)	Treatment and care (not closely specified)	Parents needed time to adjust to the new situation to be able to participate in decision-making. If doctors were using English terms to talk about the children's conditions, parents felt unable to participate in the discussion.
Janvier et al. (2019), USA, Canada, UK other	Mixed-methods study Questionnaire with open questions	To investigate how parents of children with Trisomy 13 and 18 experienced their interactions with clinicians.	Life-limiting conditions	30	332 parents (including 187 bereaved parents, 257 mothers and 74 fathers)	Limitation of treatment Treatment and care (not closely specified)	Parents felt as forced into decisions by healthcare professionals. They acted as advocates for their children. Parents who had a good relationship with healthcare professionals wanted their support and involvement in the decision-making so they wouldn't have to decide about their child’s death.
Kelly, Ganong (2011), USA	Qualitative study In-depth interviews	To explore how divorced parents make treatment decisions for their children with cancer.	Oncology	33	15 parents (8 mothers and 7 fathers	Starting treatment Trial treatment Aggressive treatment Bone-marrow transplant	Divorced parents prioritised the child's best interest over the relationship with the ex-husband/wife. Parents valued support from their new partners. Single parents lacked support from the second parent. Parents excluded stepparents from decision-making to avoid future blame for negative outcomes.
Liu et al. (2014), Taiwan	Qualitative study In-depth interviews	To explore the parental experience of making a Do Not Resuscitate decision for their child.	Life-limiting conditions Oncology Intensive care unit		16 parents (including bereaved parents, 9 mothers and 7 fathers)	Attempting resuscitation Do Not Resuscitate status	The decision about the Do Not Resuscitate status was difficult. It made parents feel responsible for the death of their child. Parents felt pressured by healthcare professionals to sign the Do Not Resuscitate form. Parents found helpful their faith in God and believed in the reincarnation of the child.
Mitchell et al. (2019), UK	Qualitative study In-depth interviews	To provide an in-depth insight into the experiences and perceptions of parents who had made end-of-life care decisions for their children.	Life-limiting conditions Intensive care unit	36	17 parents (11 mothers and 6 fathers)	Limitation of treatment End-of-life decisions Advanced care planning	Parents described their experience as difficult, as they were having conflicting emotions. While some parents wanted advanced care planning and to have information about the end-of-life, others did not want this, keeping a hope that the child would recover.
Parker et al. (2021), USA	Qualitative study Semi-structured interviews	To investigate the decision-making process of parents deciding about the enrolment of their ill child in a clinical trial.	Oncology	26	20 parents (17 mothers and 3 fathers)	Enrolment in clinical trials	Deciding whether to continue with standard or trial treatment was difficult and overwhelming for parents. Parents were worried their decision would affect their child’s future health, and they would feel guilty if the outcome were negative.
Popejoy (2015), UK	Qualitative study Semi-structured interviews	To get an understanding of the lived experience of parents throughout the process of making and revising end-of-life care decisions for their child.	Life-limiting conditions	33	3 bereaved parents (mothers only)	End-of-life decisions (place of care, place of death, limitation of treatment)	Parents valued cooperation with the medical team and passed the responsibility for the decision-making onto the physicians. The end-of-life decision was perceived as difficult or impossible as it led to the child’s death. Parents acknowledged the need to have a plan and not to make decisions in the time of crisis. Plans can be changed based on the current health condition of the child.
Rapoport et al. (2013), USA	Qualitative study In-depth interviews	To explore parental perceptions about the experience and their child’s quality of death after choosing forgoing artificial nutrition and hydration in children at the end-of-life.	Life-limiting conditions	34	11 bereaved parents (6 mothers and 6 fathers)	Forgoing artificial nutrition and hydration	Parents needed support from healthcare professionals and the medical team to have the same opinion and not contradict themselves. Forgoing artificial nutrition and hydration was a difficult decision, but parents felt at peace with it as it improved the quality of life of their child. Some parents felt judged by healthcare professionals or their family/friends for their decision.
Robertson et al. (2019), Australia	Qualitative study Semi-structured interviews	To explore parents’ and adolescents’ views and perceptions of the treatment decision-making process in paediatric oncology.	Oncology	33	25 parents (23 mothers and 2 fathers) 5 children	Clinical trial Central venous access insertion/removal Start of treatment Fertility preservation Radiotherapy	Parents experienced information overload, which made them unable to analyse all the information and make decisions. They trusted their oncologist to make the best treatment decision. They felt like they lacked the medical expertise to make the decisions by themselves and felt pressured to make decisions while not having enough information to do so. Parents believed the child should be involved in minor decisions.
Sharman et al. (2005), USA	Qualitative study Semi-structured In-depth interviews	To identify and describe factors important to parents during the decision-making process.	Life-limiting conditions Oncology	29	14 parents (9 mothers and 5 fathers)	Limitation of treatment Withdrawal of life support	Parents appreciated support from healthcare professionals while making the decision. They relied on the healthcare professionals’ expertise. They sometimes did not have enough time and felt pressured into a decision by healthcare professionals. Parents acknowledged the quality of life of the child and the child’s will to live/die and leaned their faith while making the decision. If possible, parents were including the child in the decision-making.
Stewart et al. (2012), USA	Qualitative study Semi-structured interviews	To describe the process of parents making major treatment decisions for their children with cancer.	Oncology	32	15 parents (9 mothers and 6 fathers)	Clinical trial Bone marrow transplant	Parents were determined to make the right decision for their children. They valued support from the healthcare professionals. The decisions they had to make were difficult and overwhelming. Parents experienced various emotions such as anxiety, fear, depression, stress and anger. Parents felt exhausted and had a high level of uncertainty while making the decisions. Parents relied on their personal faith in God and on spirituality.
Sullivan et al. (2020), Australia	Qualitative study Semi-structured interviews	To examine bereaved parents’ views and experiences of decision-making for their child and how they reflect and live with their decision.	Life-limiting conditions Oncology	34	25 bereaved parents (gender not specified)	End-of-life decision Limitation of treatment	End-of-life decisions were experienced as difficult. Parents were doing the best for their child, which included terminating the life support or withdrawing treatment. Parents who participated in the decision-making process were more likely to feel they made the right decision. Parents who did not participate felt regrets and were more emotionally distressed.
Woodgate et al. (2010), Canada	Qualitative study Interviews	To get a detailed, in-depth understanding of parents’ participation in decision-making about childhood cancer clinical trials.	Oncology	31	31 parents (20 mothers and 11 fathers)	Enrolment in clinical trials	The decision-making was difficult and overwhelming. The decision seemed to be impossible to make. Parents relied on the support of healthcare professionals. Their relationship with them was crucial while making the decision. Some parents felt obliged to agree with the trial so they would not upset their healthcare professionals. Parents also thought about the child’s future and the possible implications of the trial for his/her health.
Yazdani et. al. (2022), Canada	Qualitative study Semi-structured interviews	To explore parents’ and healthcare professionals’ perceptions of the experience of parents making decisions for their children with life-limiting condition.	Life-limiting conditions	30	6 parents (5 mothers and 1 father)	Treatment and care (not closely specified)	Parents experienced decisional conflict; it was difficult to foresee the outcome of their decisions. Parents acted as advocates for their children and preferred to make the decisions independently or be involved in the process. Making decisions for their children was experienced as difficult, stressful and filled with uncertainty. Parents valued support from healthcare professionals and other parents.
Zaal-Shuller et al. (2016), Netherlands	Qualitative study Semi-structured interviews	To compare the experiences of parents and physicians who were involved in the end-of-life decision-making process.	Life-limiting conditions	31	17 parents (including bereaved parents, 14 mothers and 3 fathers) 11 physicians	End-of-life decision Limitation of treatment Attempting resuscitation Do Not Resuscitate status Artificial nutrition and hydration Invasive treatment	Parents felt they were the experts on their child’s health and their opinion should be taken seriously. Parents appreciated the medical advice and support from the healthcare professionals, and some felt they lacked the medical expertise needed to make the decision. Some decisions were made under time pressure, and this was perceived as disturbing. Parents also relied on their faith.

The included studies present data from 923 parents (including 294 bereaved parents) of 757 children. The majority of parents were mothers (*n* = 665), but most studies (*n* = 24) included fathers in the sample. Included studies explored various types of decisions. The most frequent decisions were about limitation of treatment and life-sustaining treatment (see Table 3 for an overview of types of decisions).

### Synthesis

Following a narrative synthesis, 6 themes and 21 subthemes were identified. The identified themes represent the main domains of parents’ experiences with the decision-making process (Table 4). Codes for each theme are presented in Supplemental Appendix 2.

**Table 4.** Framework of the themes.

Theme	Subthemes
1. Temporal aspects affecting the experience with decision-making	*Lack of time while making the decision* *Difficulty to foresee the future*
2. Losing control of the situation	*Not having a real choice* *Being forced into the decision* *Difficulty grasping the reality*
3. Transferring the power to decide to the doctors	*Reluctance to make decision* *Transferring the responsibility to doctors* *Relying on the doctor’s expertise* *Lack of confidence and medical expertise*
4. To be a ‘good’ parent and protect the child	*Child in the centre: what is best for the child* *Advocacy for the child* *Trying everything possible*
5. The emotional state of parents	*Overall experience* *Range of emotions* *Guilt* *Feelings after*
6. Sources of support to alleviate the parental experience	*Behaviour of doctors* *Including parents in decision-making* *Having enough information* *Being supported by loved ones* *Faith*

#### Temporal aspect affecting the experience with decision-making

The first theme includes two subthemes connected to the aspect of time, which is influencing parents while making decisions.

##### Lack of time while making the decision

Parental experiences during the decision-making process were affected by the timeframe of the decision.^35,38,44,49,56,59,60,61^ Parents were often required to make decisions under time pressure and with a sense of urgency.^35,44,60,61^ Decisions made under time pressure included the Do Not Resuscitate status, an agreement to start an oncology treatment, a placement of a central access device and end-of-life decisions.^35,44,60,61^ The lack of time meant that, in some studies, parents felt like they did not have enough information to make an informed decision and they would have preferred to have more time.^35,38,44,63^ The time pressure caused anxiety and fear and was associated with disagreements and conflicts with healthcare professionals.^38,60,61^ This subtheme was interlinked with the *Being forced into the decision* subtheme.^36,38,45,48,49,61,63^ Those parents who had been given enough time to come to a decision talked about their experience peacefully.^49,56,59^ The timeframe deemed sufficient to make sound decisions varied between a few hours to a week.^49,59^

##### Difficulty to foresee the future

Parents had to make decisions which could have a long-lasting impact on their child’s quality of life, but at the same time, they struggled to comprehend the future in its complexity.^44,48,49,52–54,56,57^ Even parents of children with pre-existing life-limiting conditions found it difficult to plan for the future and to make decisions about advanced care planning as these situations were hypothetical for them, filled with uncertainty and it was difficult to imagine them happening.^52–54,57^ Additionally, some parents were worried about how their decisions will impact the child.^52,53^

The location in which decisions were made further compounded these challenges, as parents experienced difficulties in anticipating the impact of their decisions on everyday life at home when the decision was made when the child was still in the hospital environment.^44,52,56^

#### Losing control of the situation

The losing control of the situation theme refers to the parental perception of not being in charge of the decision-making process.

##### Not having a real choice

Most parents felt like they did not have a real choice.^35,37,39,41,43,48–51,54,56,63^ This was because they were not given any alternative choices to the proposed option, and the other option meant they would agree with letting the child die, or the procedure was undertaken without asking them, and they were not given a choice in the matter.^37,39,41,51,54,56^ For parents of children with cancer, the expectation was that they would follow a treatment protocol.^43,48^

##### Being forced into the decision

Some parents felt that the final decision was not their own or that they had been manipulated or even coerced into it by the clinicians^39,44,45^ or family members.^50^ Parents felt like they did not have enough information about all options available or were not involved as they wished. When making a treatment choice which did not align with the clinician’s, a minority of parents felt they lacked support or worried about disappointing the clinician.^44,49^ Parents who thought they were coerced into decision-making felt anger, bitterness and distress and they described their experience as horrific and painful.^44,49,62,63^

##### Difficulty grasping the reality

Parents struggled with the reality of the situation when they were making decisions. Often decisions were required when parents were still dealing with challenging new information about their child’s health, such as a new diagnosis, an unexpected change in the child’s health or a sudden health decline.^35,39,44,56,63^ In several studies parents were required to make decisions while not knowing what the outcome would be and whether their treatment decision would help their child or not.^48,49,52,53^ This is closely linked with the subtheme *Difficulty to foresee the future.* With some decisions, including long-term ventilation and end-of-life decisions, parents found it difficult to accept the seriousness of the situation and were in denial about the possibility their child might die in the near future.^56,57,61^ In some cases, this led to parents unintentionally passing the responsibility for the decision-making onto the healthcare professionals.^48,49,63^

#### Transferring the power to decide to the doctors

While the previous theme *Losing control of the situation* highlighted the experience of parents not being in control of the decision-making process, this theme shows that for some parents being in control is challenging and they may prefer the doctors to be in charge. Four subthemes were identified in this theme, all related to the parental experience of letting the doctors make the decision for several reasons, as presented below.

##### Reluctance to make a decision

Some parents found it difficult to accept the responsibility for making medical decisions themselves.^36,38,39,48,51,56,58,59,63^ When decisions were made, the process was deemed impossible and offensive as parents did not know what the right decision was. A minority of parents avoided making the decisions entirely,^38,56^ or did not want to be included in the decision-making process as it engendered feelings of complicity in the death of their child or concerns about being burdened with the negative outcome.^36,51^ For other parents, it was difficult to make the decisions due to the feelings of uncertainty they experienced.^52^

##### Transferring the responsibility to doctors

In several studies, parents preferred to transfer the responsibility of decision-making onto doctors entirely, particularly parents of children with life-limiting conditions other than cancer.^36,37,39,42,48,51,52,58,59,63^ By passing the responsibility onto clinicians, some parents were able to relieve themselves of future guilt feelings.^39,42^ Although this transfer was done willingly, some felt regret afterwards for letting the physician decide and questioned whether the treatment decision done by the physician was right.^39^ Two studies brought evidence that parents found it difficult to verbalise their decision and were grateful when the physician did it for them, while others felt too much pressure to make the right decision and welcomed the option to pass the responsibility onto the physicians.^58,63^

##### Relying on the doctors’ expertise

Several parents relied on the expertise of doctors and the medical team as they believed they were doing the best for their children.^37,38,42,43,48,50,57,61,63^ It was seen as important for healthcare professionals to work together as a team and to be consistent in their approach during the decision-making process.^39,50,59^ The preference was for a familiar clinician to be involved in the process.^39,60^ Additionally, trust was important as a mediator in relieving parental distress.^37,48,55,63^

##### Lack of confidence and medical expertise

A lack of medical knowledge made it difficult for parents to make decisions related to medical care; they were concerned that their decision could negatively impact their child’s health, and they lacked the confidence to make the decision.^48,51,54,60,61,63^ When combined with parents’ perceptions of their limitations, they found it difficult to contradict the clinicians’ opinion or to question the decision made by clinicians. Emotional exhaustion further compounded parental lack of confidence in decision-making.^48^ Instead, parents relied on the clinician’s expertise and advice even when they were aware that the clinicians might not be right.^51,54,63^

#### To be a ‘good’ parent and protect the child

This theme includes three subthemes highlighting the parental need to act as a parent of their child and to protect their child.

##### Child in the centre – what is best for the child

In majority of the included studies, parents stated they had the child’s best interest in mind when making the decisions, and the decisions were based on what they believed was best for their child.^41,42,46,48,49,54,56–59,60–62^ At times, this meant going against what parents wished for. The process of balancing the child’s best interests and the parents’ wishes and uncertainties about the right decision made the experience difficult.^38,39,45,49,58,61,64^ The conflict of wanting their child to live as long as possible whilst wanting to avoid additional suffering for their child was particularly challenging.^38,41,42,54,57–59,61,62^ Additionally, parents kept hope for a positive outcome even in most adverse situations.^41,55–57,61^

##### Advocating for the child

Parents often take on the role of advocates when it comes to making critical decisions.^36,38,45,48,50,52,53,56,60^ Parents firmly believe in their responsibility to make decisions which include choices related to treatment and life support.^39,45,49,56,58,62^ There was also evidence of child involvement, either through verbal expression of their wishes or nonverbal signs that indicate their desire to continue living.^38,48,49,61,63^

Parents saw themselves as experts on their children and, in situations in which they felt like they were not getting enough support from doctors, they had a strong need to protect the child.^36,38,45,48,53,60^ Parents of nonverbal children expressed their role of being a voice for their children and the need to make the decision on the children’s behalf.^52,53,60^ In some cases, parents of children with developmental delays perceived that physicians did not always treat their child with dignity and respect because of the mental impairment and felt they had to fight for appropriate care and treatment.^36,38,60^

##### Trying everything possible

When making decisions, parents wanted to try all options of treatment available or to look for treatment elsewhere, including alternative therapies and seeking a second opinion.^36,37,41–43,45,49–51,56,57,61^ This was particularly evident when making decisions about withdrawing treatment; parents needed to be sure there were no other options remaining and that they could change their decision depending on the health state of their child.^36,43,45,49,51,54,57,58,61^ Even when the condition was uncurable and clearly terminal, some parents wanted to try all possible options.^41^

#### The emotional state of parents

The emotions experienced during the decision-making process are presented in this theme. There are not stand-alone emotions, but they are closely linked to the other themes presented in this review.

##### Overall experience

The overall experience was described by many parents as overwhelming, scary, heavy, horrible, painful, gut-wrenching, horrific and emotionally exhausting.^40,48,50,52,56,57,62^ Some parents experienced inner conflict and cognitive dissonance, which then affected their ability to make decisions.^48,52,56,57^ For others, the decision-making process was a frustrating experience, especially when the decision did not lead to the expected outcome or when parents felt they were not involved in the process.^61,63^

##### Range of emotions

During the decision-making process, many parents experienced a wide range of negative emotions, including anxiety, depression, sadness, fear, nervousness, a sense of helplessness, stress and anger.^35,42,44,48,52,53,56,57,63^ Parents felt exhausted and unable to make decisions as they were experiencing informational overload and were not able to focus their minds.^48,50,57^ In some cases, anger and frustration were associated with the feeling of not being listened to or being manipulated into a decision by professionals.^44,53^

##### Guilt

Being a parent of a child with a life-limiting condition and making decisions about their healthcare was connected with the feeling of guilt.^38,39,40,45,49,51,52,58–59,61^ Parents felt guilty for multiple reasons, including not being active in the decision-making process; letting the doctor decide; making decisions which could cause the death of their child; giving up on the child and undermining their child’s will to live. Additionally, parents were anxious that their decisions would make them feel guilty in the future, and this made it more difficult for them to participate in the decision-making process.^40,51,52^

##### Feelings after

After the decision-making process, parents experienced feelings of disappointment, helplessness and relief. Some parents experienced regret and had difficulty accepting the decision they had made.^37,38,39,45,49,50,61,62,63^ Having doubts about their decision was enhanced by feelings of uncertainty about the child’s condition, and the selected treatment approach.^48,50,59^ Nevertheless, some parents were at peace with their decision and were not experiencing regret.^37,41^

#### Sources of support to alleviate the parental experience

The last theme identifies various sources of support which can mitigate the complexity of the decision-making process and have a positive impact on the parental experience.

##### Behaviour of doctors

Parents appreciated supportive behaviour from clinicians, which included giving hope; respecting parents’ choices; being personal; and being non-judgmental.^36,40,42,48–50,52,57,59^ Doctors who were empathic, compassionate, respectful, honest, truthful and upfront, who spent time explaining the situation and gave parents time to ask questions, and those who offered options to choose from were appreciated.^36,38,40,48,50,61,63^ In contrast, parents who felt like they did not have enough support from the healthcare professionals experienced stress and felt like they had to defend their decisions.^36,44,56^

##### Including parents in decision-making

In several studies parents valued being part of the decision-making process, particularly being acknowledged and listened to by physicians and enabled to make decisions together with them.^36,38,43,48,50,52,53,56,57,60,63^ The experience of decision-making was less stressful if parents were engaged in the process, given professional guidance, treated with respect and received support from clinicians.^36,38,50,52,57,60,63^

##### Having enough information

Having sufficient information was particularly emphasised as important in the active participation in the decision-making process.^38,40,42,50,52,55–57,63^ This enabled parents to know about the options available and to trust their feelings and instincts during the process.^36,39,52,56^ The lack of information had a negative impact on parental ability to participate in the process, but finding the right amount of information was challenging as being overwhelmed with information led to similar outcomes.^38,40,56,63^ In some studies parents used other sources of information, including other parents in a similar situation and the internet.^38,40,50,52,57^

##### Being supported by loved ones

When making decisions, parents valued the support of their partner, wider family and friends.^37,38,40,46,48,50,52,56,58,59^ Support between spouses was experienced as crucial; single or divorced parents described the decision-making process as a hard task which was full of doubt given they had no spouse to discuss their decision with.^38,46,59^

##### Faith

Religiosity and faith in God had an impact on the experience with decision-making.^36,37,38,41,48,50,56,60,61^ Religious parents trusted in God’s guidance to make the right decision, and in some cases, they put the responsibility in God’s hands.^36,38,48,56,61^ Some parents believed they would meet their child in the afterlife.^41^ Praying and believing in God gave parents the strength to deal with the situation and some sense of comfort and peace.^38,48,56^

## Discussion

The purpose of this systematic review was to explore how parents experience the process of making decisions about medical care for their children with life-limiting conditions.

The review identified that participation in the decision-making process is emotionally challenging. The wide range of negative emotions experienced by parents compounds the experience by affecting their ability to make decisions and to be in control of the process. This presented review extends the knowledge of decision-making in the medical environment by providing evidence that decision-making is experienced similarly by parents, irrespective of the child’s diagnosis. This supports findings of previous research on decision-making done in a general paediatrics setting.^19,20,65,66^ It is not surprising that positive emotions were not mentioned in studies included in this review, given the lack of positive emotions described in a wider body of literature in this field.^20,65^ Interestingly, this review identified guilt, including anticipatory guilt, as an emotion frequently experienced by parents while making decisions. This finding offers a new view on guilt as the concept of guilt is usually connected with loss and bereavement^67,68^ or with the sense of responsibility for the child’s condition and suffering.^25,69^ Guilt in connection to decision-making was mentioned in previous studies with parents of preterm infants or children with disabilities^8,21^ while anticipatory guilt was described in situations when parents imagined their life after the death of the child.^69^

This review shows that parents are required to make difficult decisions in challenging circumstances, which can impact their ability to make decisions. Parents may rely on doctors to make decisions instead.

Experiencing pressure and coercion from healthcare professionals during the decision-making process was connected with negative emotions. The use of persuasive strategies by healthcare professionals when making decisions for children with life-limiting conditions was identified in a recent study by Popejoy et al.,^70^ which shows that healthcare professionals use persuasion based on their moral work done during decision-making. This presented review extends this knowledge by adding evidence that persuasive strategies can have negative impact on the emotional state of the parents. Persuasive techniques used by healthcare professionals include presenting preferred options in a more positive light while not presenting other options as viable by healthcare professionals.^15,70^

Being required to make decisions in a limited period of time was experienced as stressful and, in some cases, led to conflicts with healthcare professionals. In previous research, time was identified as the main environmental barrier to shared decision-making.^24,65,66,71^ The timeframe in which the decision took place was found to directly affect the parental ability to participate in the decision-making and their perception of being pushed into the decision.^24,65,66,71^ The findings of this review shows that parents needed to have enough time to process information provided by the physicians in order to make informed decisions, a finding congruent with previous research.^24^

This review identified that parents need to keep their parental role, to be a ‘good parent’, and to act as an advocate for their child during the decision-making process. The need to act as a ‘good parent’ represents an interesting concept explored in previous research^72^ and is characterised by making informed decisions based on the child’s best interest, being responsible for the decisions, advocating for the child and protecting the child from suffering.^19,73,74^ The findings from this review bring new insight by collating available evidence that this attitude puts parents in a difficult position as they try to balance their own wishes and uncertainties with the need to be a ‘good parent’ when making decisions for their child. This conflict between their own desires and what is best for the child can put additional strain on parents and negatively affect their communication about medical care with healthcare professionals.^22^ To guide their decisions, parents used their subjective perception of the child’s will to live. This was described in a previous study, where the child’s will to survive affected parental decision-making.^8^

This systematic review identified that limited medical knowledge, other parental insecurities and emotional exhaustion led to a lack of confidence in parents about their ability to act on behalf of their children. While this finding is consistent with previous studies, which found that parental belief about their deficit in medical knowledge had a negative impact on their involvement in the decision-making process,^20,24,66^ this review shows that parents may follow the decisions made by healthcare professional even when they do not agree with them. Parental ability to make decisions is further affected by the situation and circumstances in which the decision-making took place and by the emotional state of parents, including the feeling of being stressed, overwhelmed or in shock.^22,75^

Participation in decision-making is extremely stressful for parents, but this review has found that it is possible to mitigate their negative experience.

The support provided by a spouse, family or friends can positively impact the experience with decision-making. This finding is consistent with a previous systematic review set in paediatric palliative care, in which friends and family were identified as an important source of support during end-of-life care, easing parental feelings of guilt and doubt.^25^ This presented review highlights that single parents who lacked support from a spouse experienced additional challenges as they were required to make decisions on their own. This is a poorly explored area and future research should focus on this population.

Another strategy parents used during decision-making was their faith in God and praying, which is consistent with findings about /related to the importance of faith in decision-making identified in previous research in paediatric medicine.^20,24,25,76^ In this review, trust in God’s guidance and parents’ belief that they will meet their child in afterlife helped parents to find a sense of comfort, hope and peace. Similarly, Hexem and Tan^25,76^ identified the benefits of using religion and faith by parents during decision-making. The potential of Church and religious communities as sources of support for parents, reported in the study by Hexem et al.,^76^ was not identified in this review.

The experience was greatly affected by the behaviour of healthcare professionals. Enabling parents to keep their hope and respecting their parental role made the experience less traumatic. Parents value honest communication and being listened to, as highlighted in previous research.^23,25,75^ The role of clinicians was found to affect the ability of parents to participate in the decision-making process, which is consistent with findings of how the behaviour of clinicians can influence parental involvement in decision-making.^4,66^ Parents perceived their experience as less stressful when/if they were able to actively engage in the decision-making. To do so, they needed to be invited by the healthcare professionals, as the power distribution in the medical setting is not balanced, and it can be difficult for parents to engage in the decision-making process.^15,66,77^

Having adequate information was identified in this review as a prerequisite for parents’ active participation, which is consistent with findings of previous research focussed on the parental need to have enough information to be able to engage in the decision-making process.^4,66,75,78^

The findings of this review suggest that healthcare professionals involved in care of children with life-limiting conditions can make the experience of parents with decision-making less traumatic by actively inviting parents to participate in the decision-making, respecting their role as parents and giving them enough information.

## Strengths and limitations

This review has several limitations. The use of narrative approach enabled the authors of this review to include methodologically heterogeneous studies, which was challenging for the subsequent synthesis. The inclusion criteria were not limited to a specific diagnosis; therefore, a larger number of studies were included in the review, thus possibly affecting the robustness of the synthesis. The data extraction and analysis were conducted by one reviewer, which could lead to a personal bias in the data interpretation. Due to limited resources, only studies written in English and Czech were eligible for the review. Although the included studies originated from several countries, the impact of different cultures was not explored in this review as it was not the focus of the review. Future research in this field should explore the impact of cultural settings on decision-making in paediatrics. The participants in the studies included in this review were predominantly mothers. Whilst the fathers’ experiences were included, there is a paucity of research about the paternal experience. Additionally, the studies were retrospective in nature, and some included bereaved parents, which could have affected parents’ recollection of their experience.

Notwithstanding the limitations listed above, this review has several strengths. To our knowledge, this is the first review focussed solely on parental experiences of decision-making for their children with life-limiting and life-threatening conditions. This review provides a robust synthesis of available evidence of the studied phenomenon. Wider inclusion criteria made it possible to include studies focussed on different types of diagnoses of the children and on various types of decisions. This approach made it possible to get an understanding of the experience from a wide perspective. By using a narrative approach, it was possible to synthesise the data without delineating between different types of decisions and diagnoses. Although the data extraction and analysis were done by one reviewer, the whole process was supervised by the other authors, including the screening of eligible studies, the development of preliminary and final themes and discussion of the findings. Each of the included studies was assessed for its quality by two reviewers, although studies were not excluded based on the score achieved.

## Conclusion

This study brings evidence that parental experience with decision-making represents a complex phenomenon. The experience with decision-making was not affected by the conditions of the child, which suggests that this is a universal experience framed by the parental role. Clinicians need to be aware of how parents experience their participation in the process and provide them with adequate support. Parents should be actively invited and encouraged to participate in the decision-making by clinicians. Considering the long-lasting impact this experience has on parents, it is necessary to tailor the level of participation individually for each parent and enable them to engage at their preferred level. Further research should focus on the experience of fathers and single parents, as this population is understudied and on exploring decision-making in various cultural contexts.

## Supplemental Material

sj-pdf-1-pmj-10.1177_02692163231214414 – Supplemental material for Parents’ experiences of being involved in medical decision-making for their child with a life-limiting condition: A systematic review with narrative synthesis Click here for additional data file. Supplemental material, sj-pdf-1-pmj-10.1177_02692163231214414 for Parents’ experiences of being involved in medical decision-making for their child with a life-limiting condition: A systematic review with narrative synthesis by Kristyna Polakova, Faraz Ahmed, Karolina Vlckova and Sarah G Brearley in Palliative Medicine

sj-pdf-2-pmj-10.1177_02692163231214414 – Supplemental material for Parents’ experiences of being involved in medical decision-making for their child with a life-limiting condition: A systematic review with narrative synthesis Click here for additional data file. Supplemental material, sj-pdf-2-pmj-10.1177_02692163231214414 for Parents’ experiences of being involved in medical decision-making for their child with a life-limiting condition: A systematic review with narrative synthesis by Kristyna Polakova, Faraz Ahmed, Karolina Vlckova and Sarah G Brearley in Palliative Medicine
