# Supplementary material for: Parents’ experiences of being involved in medical decision-making for their child with a life-limiting condition: A systematic review with narrative synthesis
Source: Palliat Med. 2023 Dec 6;38(1):7–24. doi: 10.1177/02692163231214414 (PMC10798032; doi:10.1177/02692163231214414)
Supplement: sj-pdf-2-pmj-10.1177_02692163231214414 – Supplemental material for Parents’ experiences of being involved in medical decision-making for their child with a life-limiting condition: A systematic review with narrative synthesis [file sj-pdf-2-pmj-10.1177_02692163231214414.pdf]

Appendix 2

Codes

**Theme**	**Subthemes**	**Codes**
Temporal aspects affecting the experience with decision-making	Lack of time while making the decision<br>Difficulty to foresee the future	Lack of time<br>Difficult to plan for the future
Losing control of the situation	Not having real choice<br>Being forced into the decision<br>Difficulty grasping the reality	Being forced into decision<br>Difficulty to grasp reality<br>Feeling not making the decision by themselves<br>Being judged<br>Conflict with doctors<br>Information overload<br>Not respecting parents<br>Not having real choice
Transferring the power to decide to the doctors	Reluctance to make decision<br>Transferring the responsibility to doctors<br>Relying on the doctor´s expertise<br>Lack of confidence and medical expertise	Don´t want to make the decision<br>Lack of confidence<br>No medical expertise<br>Previous experience<br>Transferring the responsibility to HCP<br>Doctor´s expertise

To be a "good" parent and protect the child	Child in the centre-what is best for the child<br>Advocacy for the child<br>Trying everything possible	Taking responsibility for the decision<br>Advocacy<br>Including the child<br>Keeping hope<br>Keeping all options open<br>What is best for the child<br>Making the right decision<br>Trying all options of treatment<br>Outcome of the child<br>Quality of life<br>Keeping ole of a parents<br>Abandoning the child<br>Protecting the child<br>Asking second opinion<br>Trying all options of treatment<br>Making difficult decisions
The emotional state of parents	Overall experience<br>Range of emotions<br>Guilt<br>Feelings after	Being exhausted<br>Difficult decision<br>Frustration<br>Guilt feeling<br>Regret about the decision<br>Emotions after<br>Having doubts<br>Overwhelming<br>Conflicts<br>Anger<br>Information overload

Sources of support to alleviate the parental experience	Behaviour of doctors<br>Including parents in decision-making<br>Having enough information<br>Being supported by loved ones<br>Faith	Faith and praying<br>Having enough information<br>Cooperation with parents<br>Lack of support<br>Respecting/not respecting parents<br>Support from doctors<br>Trust<br>Specialist work as team<br>Support from family<br>Enough information<br>Respecting parents’ choices even if different<br>Communication<br>Telling truth
